# The effect of addition of 2DCT scans and 3DCT scans for the classification of tibial plateau fractures: a systematic review

**DOI:** 10.1007/s00068-023-02344-3

**Published:** 2023-09-28

**Authors:** Jellina Mariska Huitema, Nynke van der Gaast, Ruurd Lukas Jaarsma, Job Nicolaas Doornberg, Michael John Richard Edwards, Erik Hermans

**Affiliations:** 1 Department of Trauma Surgery, Radboud University Medical Center, Radboud University, Geert Groteplein Zuid, 6525 GA Nijmegen, The Netherlands; 2 Department of Orthopaedic and Trauma Surgery, Flinders Medical Centre, Flinders University, Adelaide, Australia; 3 Department of Orthopaedic Surgery, University Medical Center Groningen, Groningen, The Netherlands

**Keywords:** Tibial plateau fractures, Interobserver agreement, Schatzker classification, AO/OTA classification, Luo's three-column concept

## Abstract

**Purpose:**

In this systematic review, we evaluate the effect of radiographs and 2D and 3D imaging techniques on the interobserver agreement of six commonly used classification systems for tibial plateau fractures.

**Methods:**

In accordance with PRISMA guidelines, PubMed, Cochrane, Embase and Web of Science were searched for studies regarding the effect of 2D and 3D imaging techniques on the interobserver agreement of tibial plateau classification systems. Studies validating new classification systems, not providing own data or only providing information on the interobserver agreement for radiographs were excluded. Studies were scored based on the ROBINS-I risk of bias tool.

**Results:**

Our review analysed 14 studies on different classification systems used for tibial plateau fractures in clinical practice, with the Schatzker classification being the most commonly used classification system. The results showed that the addition of 2D CT led to a significant improvement of interobserver agreement for one study. However, other included studies showed varying levels of interobserver agreement, ranging from fair to substantial according to the interpretation by Landis and Koch. The addition of 3D CT resulted in a significant deterioration in one study for the Schatzker classification. Similar to the addition of 2D CT, the interobserver agreement for the Schatzker classification with the addition of 3D CT were heterogeneous ranging from fair to almost perfect according to the interpretation by Landis and Koch.

**Conclusions:**

The use of 2D CT can be recommended for classifying tibial plateau fractures with the Schatzker classification, AO/OTA classification and Hohl classification. The value of 3D CT on the interobserver agreement of commonly used classification systems remains uncertain and unproven. Therefore, we do not recommend the use of 3D CT for the classification of tibial plateau fractures. Overall, the advancement of imaging techniques is not in line with the advancement in interobserver agreement on fracture classification.

**Supplementary Information:**

The online version contains supplementary material available at 10.1007/s00068-023-02344-3.

## Introduction

The classification of fractures has traditionally been part of fracture assessment and fracture treatment. Where classification systems initially were descriptive and based on the appearance of the limb, with the advent of radiographs, classifications were soon based on specific location and extent of displacement [1, 2]. For example in tibial plateau fractures, the Hohl classification [2, 3] and the Schatzker classification [4] describe respectively four and six different types of tibial plateau fractures according to the fracture morphology observed on radiographs. The Hohl classification [2, 3] is distinguishing between undisplaced, local depression, total depression and split fractures, while the Schatzker classification [4] further expanded with incorporating fracture location—medial, lateral or bicondylar—along with morphology. More comprehensive classification systems such as the AO/OTA classification [5] and the Duparc and Ficat classification [6] both consist up to sixteen subtypes. The AO/OTA classification [5] encompassed partial articular fractures and complete articular fractures, while the Duparc classification gained recognition for its inclusion of posteromedial tibial plateau fractures.

With the introduction of computed tomography (CT), an axial view of the tibial plateau became available and tibial plateau fractures now appeared more complex than radiographs could depict. New classification systems based on the axial view of the two-dimensional (2D) CT scan were created. For example Luo’s three column concept [7], where the tibial plateau is divided in a lateral, medial and posterior column. Moreover, the ten segment classification has been introduced based on a 3D articular surface reconstruction of the tibial plateau and divides the anterior and posterior tibial plateau into five different segments, which could guide surgeons in their surgical approach [8]. Lastly, in 2018, the Schatzker classification was revised by Kfuri et al. [9]. The 3D extension of this commonly used classification system adds a new method of notation providing extra details of the injury and a clear guideline for preoperative planning to prevent surgical errors and improve treatment outcomes.

The effect of CT techniques on the reliability of classification systems has been extensively studied and their additional value can still be questioned [10–12]. For tibial plateau fractures, Millar et al. [13] provided a comprehensive analysis of the reliability of classification systems based on studies published until October 2016, and showed that five classification systems were tested for inter- and intra-observer reliability: AO/OTA [5], Schatzker et al. [4], Duparc and Ficat [6], Hohl[3], and Luo et al. [7]. Interobserver agreement for these classification systems ranged from fair to substantial based on radiographs, with the simpler classification systems frequently achieving a higher interobserver agreement. This variable effect of different imaging modalities on the interobserver agreement is also shown in studies of proximal humerus fractures [14, 15] and distal radius fractures [16, 17]. Therefore, to better understand the effect of different imaging modalities on the interobserver agreement of tibial plateau fracture classification systems, we aim to answer the following questions:

1. What is the effect of adding 2D CT on the interobserver agreement of the AO/OTA classification, Schatzker classification, Duparc and Ficat classification, Hohl and Luck classification, Luo’s (revised) three-column concept and the 10-segment classification?
2. What is the effect of adding 3D CT on the interobserver agreement of the AO/OTA classification, Schatzker classification, Duparc and Ficat classification, Hohl and Luck classification, Luo’s (revised) three-column concept and the 10-segment classification?

## Methods

The protocol of this systematic review is registered in the International prospective register of systematic reviews (PROSPERO) with record number CRD42020211877. This study was conducted in accordance with PRISMA guidelines. [18]

### Search strategy and study selection

A search strategy for each electronic database was created with help from a professional medical librarian (Suppl Appendix 1). The following electronic databases were independently searched by two authors (JH and NG): PubMed, Cochrane, Embase and Web of Science. The search was last updated on the 5th of May 2022. Inclusion criteria were studies who reported interobserver agreement for the classification of any tibial plateau fractures comparing 2D CT and/or 3D CT using different classification systems. The studies that used the following classification systems that were included: Schatzker classification, AO/OTA classification, Duparc classification, Hohl classification, Luo’s (revised) three-column concept and the 10-segment classification. Exclusion criteria were: case reports, animal studies, conference abstracts, studies on paediatric fractures, open fractures and studies only providing information on the interobserver agreement for radiographs or studies purely reviewing the implementation of a new classification system. Additionally, studies in languages other than English, French or German, were excluded. The results of literature search were imported into the software Endnote X9.2. After duplicates were removed, two authors (JH and NG) independently assessed the studies for eligibility based on title and abstract. All potentially relevant articles were retrieved in full-text and re-assessed before final inclusion. Quality assessment was performed by using the ROBINS-I criteria for non-randomized studies [19]. Any disagreement was resolved in discussion with the senior author (EH).

### Extraction of data

Two authors (JH and NG) extracted the data from all included studies. The extracted data consisted of the following items: list of authors, publishing year, used imaging methods, used classification systems, and interobserver agreement of the classification systems.

### Statistical analysis

For the interpretation of the interobserver agreement, we used the division recommended by Landis and Koch [20]; a kappa between 0.01 and 0.20 reflects slight agreement, between 0.21 and 0.40 reflects fair agreement, between 0.41 and 0.60 reflects moderate agreement, between 0.61 and 0.80 reflects substantial agreement, and greater than 0.81 reflects almost perfect agreement. A difference in interobserver agreement was considered significant when upper and lower boundaries of 95% confidence intervals did not overlap or when a *p *value < 0.05 was stated.

## Results

### Inclusion of studies

**Fig. 1:**
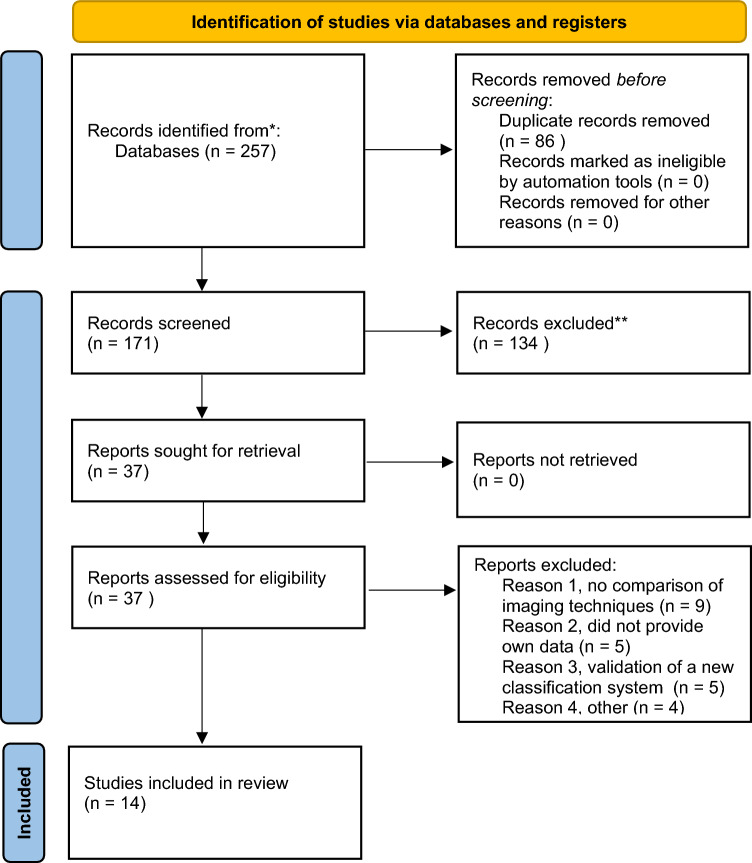
PRISMA flow diagram

257 publications were identified through PubMed (*n* = 71), Embase (*n* = 44), Cochrane library (*n* = 80), and Web of Science (*n* = 62). After excluding duplicates (*n* = 86), 171 articles were screened for relevance based on title and abstract. 134 articles were excluded, and the remaining 37 articles were full text screened. Twenty-three articles did not meet our eligibility criteria due to the following reasons: no comparison of imaging techniques (*n* = 9); did not provide own data (*n* = 5); validation of different classification systems (*n* = 5); other (i.e. providing categorical data, reviewing other imaging methods) (*n* = 4). Ultimately, 14 studies were included. Details of the selection process is shown in the PRISMA flow diagram [21] (Fig. 1).

### Risk of bias assessment

Based on the ROBINS-I risk of bias tool, the overall predicted direction of bias was varying between a critical risk of bias to low risk of bias for all included studies. Five studies scored a low risk of bias, Six studies scored moderate, one study scored serious, and two studies were considered to have a critical risk of bias. Bias category 6—bias in measurement of outcomes—of the ROBINS-I risk of bias tool was considered not applicable for our review and was excluded in the assessment (Table 1).

**Table 1:** Overview of the risk of bias assessment according to the ROBINS-I risk of bias tool

	Part 1: bias due to confounding		Part 2: bias in selection of participants into the study		Part 3: bias in classification of interventions		Part 4: bias due to deviations from intended interventions		Part 5: bias due to missing data		Part 6: bias in measurement of outcomes		Part 7: bias in selection of the reported result		Overall
Brunner (2010)	Low		Low		Low		Low		Low		N/A		Moderate		Moderate
Castiglia (2018)	Low		Low		Moderate		Low		Low		N/A		Low		Moderate
Chan (1997)	Critical		Low		Moderate		Low		Low		N/A		Low		Critical
de Lima Lopes (2014)	Low		Low		Low		Low		Low		N/A		Low		Low
Doornberg (2011)	Low		Low		Low		Low		Low		N/A		Moderate		Moderate
Gicquel (2013)	Low		Low		Low		Low		Low		N/A		Low		Low
Hu (2009)	Low		Low		Low		Low		Low		N/A		Low		Low
Masouros (2022)	Low		Low		Moderate		Low		Low		N/A		Low		Moderate
Mellema (2016)	Serious		Low		Low		Low		Serious		N/A		Low		Serious
Patange Subba Rao (2014)	Low		Low		Low		Low		Low		N/A		Moderate		Moderate
Taşkesen (2017)	Low		Low		Low		Low		Low		N/A		Low		Low
te Stroet (2011)	Critical		Low		Low		Low		Low		N/A		Low		Critical
Van den Berg (2020)	Low		Low		Low		Low		Low		N/A		Low		Low
Yao (2022)	Low		Low		Moderate		Low		Low		N/A		Low		Moderate

### Evaluation of use of classification systems of included studies

The Schatzker classification was the most commonly used classification system, with 13 out of the 14 included studies assessing this classification system. Moreover, seven studies assessed the interobserver agreement of the AO/OTA classification; three studies assessed the Hohl classification; two studies assessed the Duparc classification and the ten segment classification was only used in one study.

The study design and the reported interobserver agreement per classification system and per imaging modality of each included study are summarized in Tables 2 and 3.

**Table 2:** Overview of the interobserver agreement and study characteristics of studies focusing on 2D imaging techniques

Study	Classification systems	Average interobserver agreement			
			Rx	Rx + 2DCT	2DCT
Brunner (2010)	AO/OTA	κ_AO/OTA_	0.43*	0.73*	
	Schatzker	κ_schatzker_	0.42*	0.76*	
	Hohl	κ_Hohl_	0.43*	0.77*	
Castiglia (2018)	Schatzker	κ_schatzker_	0.58	0.62	
Chan (1997)	Schatzker	κ_schatzker_	0.62	0.61	
de Lima Lopes (2014)	Schatzker	κ_schatzker_	0.36	0.35	
Gicquel (2013)	AO/OTA	κ_AO/OTA_	0.36^†^	0.48^†^	
	Schatzker	κ_schatzker_	0.40^†^	0.48^†^	
	Duparc	κ_duparc_	0.37^†^	0.47^†^	
Masouros (2020)	AO/OTA	κ_AO/OTA_	0.20^†^	0.23^†^	
	Schatzker	κ_schatzker_	0.36^†^	0.36^†^	
	Luo	κ_Luo_	X	0.50	
te Stroet (2011)	Schatzker	κ_schatzker_	0.47	0.46	
Taşkesen (2017)	AO/OTA	κ_AO/OTA_	0.43		0.54
	Schatzker	κ_schatzker_	0.51		0.61
	Luo	κ_Luo_	X		0.47
	Hohl	κ_Hohl_	0.45		0.51
	Duparc	κ_duparc_	0.39		0.52

*Difference in interobserver agreement is significant

^†^Difference in interobserver agreement is not significant

For other values, no information on significance was available

**Table 3:** Overview of the interobserver agreement and study characteristics of studies focusing on 3D imaging techniques

Study	Classification systems	Average interobserver agreement							
			Rx	Rx + 2DCT	Rx + 2DCT + 3DCT	Rx + 3DCT	2DCT	2DCT + 3DCT	3DCT
Castiglia (2018)	Schatzker	κ_schatzker_	0.58	0.62	0.64				
Doornberg (2011)	AO/OTA	κ_AO/OTA_		0.54^†^	0.55^†^				
	Schatzker	κ_schatzker_		0.55^†^	0.60^†^				
	Hohl	κ_Hohl_		0.67^†^	0.75^†^				
Hu (2009)	AO/OTA	κ_AO/OTA_		0.71		0.83			
	Schatzker	κ_schatzker_		0.74		0.85			
Mellema (2016)	Schatzker	κschatzker					0.37*	0.29*	
	Luo	κLuo					0.31	0.25	
Patange (2013)	Schatzker	κ_schatzker_		0.54^†^	0.55^†^				
	Luo	κ_Luo_		0.72^†^	0.87^†^				
Van den Berg (2020)	Revised Luo	κ_revisedLuo_					0.48		0.43
Yao (2022)	AO/OTA	κ_AO/OTA_		0.56					0.59
	Schatzker	κ_schatzker_		0.64					0.66
	Revised Luo	κ_revisedLuo_		0.53					0.65
	Ten- segment	κ_tensegment_		0.60					0.73

*Difference in interobserver agreement is significant

^†^Difference in interobserver agreement is not significant

For other values, no information on significance was available

### Evaluation of the addition of 2D CT

Eight studies evaluated the influence of 2D CT on the interobserver agreement for five classification systems. Brunner et al. [22] showed a significant improvement in interobserver agreement when comparing radiographs to radiographs and 2D CT for the AO/OTA, Schatzker, and Hohl classification. Furthermore, Taşkesen et al. [23] and Castiglia et al. [24] and te Stroet et al. [25], showed an improvement in interobserver agreement; however, no additional information on significance was given. The studies of Chan et al. [26] and de Lima Lopes et al. [27] showed a minor deterioration in interobserver agreement for the Schatzker classification. Unfortunately, similar to the previous mentioned studies, no additional information on significance was provided. The studies of Gicquel et al. [28] and Masouros et al. [29] did provide a 95% confidence interval, although, no significant changes in interobserver agreement were described. A substantial agreement was the highest achieved interobserver agreement for 2D CT and was achieved in the study of Brunner et al. [22]. All reported kappa values for each classification system are summarized in Table 2.

### Evaluation of the addition of 3D CT

Seven studies evaluated the influence of 3D CT on the interobserver agreement for the AO/OTA, Schatzker, Hohl and Luck classification, Luo’s (revised) three column concept and the 10-segment classification. First, the study of Mellema et al. [30] compared 2D CT with the combination of 2D and 3D CT and showed a significant deterioration for the Schatzker classification after the addition of 3D CT. Moreover, van den Berg et al. [31] compared 2D CT with 3D CT solely and showed a deterioration in interobserver agreement after addition of 3D CT, they did not provide information on significance. Doornberg et al. [32] and Patange et al. [33] compared radiographs and 2D CT to the combination of 3D CT, 2D CT and radiographs, but both studies showed no significant differences. Castiglia et al. [24] compared radiographs with the combination of radiographs, 2D CT and 3D CT and showed a small, categorical improvement in interobserver agreement; however, no information on significance was given. Hu et al. [34] compared radiographs and 2D CT with the combination of radiographs and 3D CT, and showed that the change of 2D CT to 3D CT resulted in an almost perfect interobserver agreement. Again, like other studies, no additional information on significance was given. In contrast, Yao et al. [35] showed no significant difference in interobserver agreement when comparing 3D CT with the combination of 2D CT and radiographs. An almost perfect interobserver agreement was the highest achieved agreement and was achieved in the study of Hu et al. [34]; however, this value was not significant. All reported kappa values for each study are summarized in Table 3.

## Discussion

In this study we aimed to review the influence of radiographs, 2D CT and 3D CT on the interobserver agreement for six different classification systems for tibial plateau fractures and illustrates the complexity of tibial plateau fractures and it is fracture classification. The most commonly used classification system in clinical practice is the Schatzker classification, which was reflected in our review with 13 out of 14 studies addressing this classification system. Results showed a significant improvement after the addition of 2D CT for one study. However, the addition of 2D CT resulted in heterogeneous values of interobserver agreement from a fair to substantial agreement according to the interpretation by Landis and Koch. The addition of 3D CT did result in a significant deterioration for one study for the Schatzker classification. Similar to the addition of 2D CT, the addition of 3D CT resulted in heterogeneous values of interobserver agreements for the Schatzker classification ranging from fair to an almost perfect agreement.

Nowadays, an additional CT scan after conventional radiography is highly recommended for tibial plateau fractures. For, this examination can give significantly more information about the fracture characteristics and can improve preoperative assessment [36, 37]. However, there is a discrepancy between the improvement of imaging methods and the interobserver agreement on classification systems; our systematic review showed that the interobserver agreement for different classification systems was widely varying for 2D CT (range 0.35–0.77). The study of Brunner et al. [22] showed a significant improvement for the Schatzker classification, the AO/OTA classification and the Hohl classification. Moreover, this study stated that after addition of 2D CT, the observers indicated no difficulties classifying, in contrast to classification based on radiographs only. Furthermore, Castiglia et al. [24] showed that additional 2D CT imaging changed the surgical approach for tibial plateau fractures (Schatzker type II, V and VI) significantly. This suggests that despite improvement in interobserver agreement not always seen, the addition of 2D CT can improve understanding of fracture patterns and surgical approach. Therefore, we can recommend the use of 2D CT for the classification of tibial plateau fractures for the Schatzker classification, AO/OTA classification and Hohl classification.

3D CT is a general accepted imaging technique that provides a realistic and detailed view of an intra-articular fracture. Despite the assumption that 3D CT provides more information on fracture morphology and therefore increases fracture understanding, our systematic review showed widely varying values of interobserver agreement after the addition of 3D CT for fracture classification (range 0.25–0.87). One study resulted in a significant deterioration of interobserver agreement after the addition of 3D CT for fracture classification. Based on the significant results in this study and the heterogeneous results reported in this review, the effect of 3D CT for the classification systems remains questionable. Therefore, we do not recommend the use of 3D CT for the classification systems used in this review.

This study shows that there are a wide range of classification systems for tibial plateau fractures and that the classification is a complex task for surgeons. The discrepancy between the advancement of imaging techniques and the advancement of interobserver agreement in classification systems for tibial plateau fractures is also seen for other intra-articular fractures. A systematic review on the influence of imaging methods on the reliability of proximal humerus classification systems demonstrated in two studies that the interobserver agreement for 2D CT was higher than for 3D CT; however, two other studies stated the exact opposite [38]. This discrepancy could be caused by the acquired preferences and values of surgeons during their surgical training and their work experience: they will focus on their most valued characteristics and other, less valued characteristics will remain unrecognized. This complicates classification of complex fractures with multiple fracture characteristics and could partly cause the variation of interobserver agreement reported in our systematic review.

There were several limitations in this systematic review: (1) ideally, a meta-analysis is performed to evaluate the effect of imaging techniques on the interobserver agreement. However, among the 14 studies included, ten studies did not provide information on the standard error and/or confidence interval in their analysis. We tried to contact the corresponding authors for this additional information. Unfortunately, no additional data was provided. This absence of essential information, combined with the use of different classification systems, prohibited a direct comparison of the included studies using a meta-analysis. Additionally, due to the limited number of studies addressing significance, it was not feasible to perform a comprehensive analysis of heterogeneity across the included studies [39]. Nevertheless, the included studies exhibit methodological diversity, particularly regarding the number of fractures and observers to calculate the interobserver agreement. One could argue that an increase in the number of observers and subclassifications within different classification systems could lead to a decreased interobserver agreement. For example, the study of Mellema et al. [40] involved the largest number of observers (*n* = 81) assessing 15 tibial plateau fractures and showed the lowest levels of interobserver agreement for both 2D CT and 3D CT. In contrast, the study by Hu et al. [34] had the fewest number of observers (*n* = 4) and reported the highest levels of interobserver agreement, reaching an almost perfect agreement for the Schatzker classification and AO/OTA classification. (2) This study focusses on six different classification systems, whereas there is a wider variety of classification systems for tibial plateau fractures. We did address classification systems based on radiographs as well as 2D CT and 3D CT to provide a broad overview of different classification systems. However, only the study of Yao et al. [35] addressed the ten-segment classification and no studies have assessed the interobserver agreement for the revised Schatzker classification. Therefore, this review does not allow for any conclusions regarding these classification systems. (3) None of the included studies studied the effect of level of observer experience on interobserver agreement. Therefore, this systematic review did not allow for further specification towards the level of experience of surgeons, potentially allowing for more heterogenous results. In our previous study [41], we evaluated the additional effect of 3D print models and showed that junior residents improved the most in terms of interobserver agreement. Similar results were seen for the effect of 3D printed models and 3D virtual reality on acetabular fractures, where junior residents and interns improved most, compared to consultant orthopaedic surgeons. [42, 43]

## Conclusion

The use of 2D CT can be recommended for classifying tibial plateau fractures with the Schatzker classification, AO/OTA classification and Hohl classification. The value of 3D CT on the interobserver agreement of commonly used classification systems remains uncertain and unproven. Therefore, we do not recommend the use of 3D CT for the classification of tibial plateau fractures. Overall, the advancement of imaging techniques is not in line with the advancement in interobserver agreement on fracture classification.

### Supplementary Information

Below is the link to the electronic supplementary material.

Supplementary file1 (PDF 115 KB)

## Data Availability

The data utilized for this systematic review is available upon reasonable request.
