# Supplementary material for: The effect of addition of 2DCT scans and 3DCT scans for the classification of tibial plateau fractures: a systematic review
Source: Eur J Trauma Emerg Surg. 2023 Sep 28;50(1):71–9. doi: 10.1007/s00068-023-02344-3 (PMC10923987; doi:10.1007/s00068-023-02344-3)
Supplement: Supplementary file 1 — Supplementary file1 (PDF 115 KB) [file 68_2023_2344_MOESM1_ESM.pdf]

Search strategy ‘The use of radiographs, 2D and 3D CT scans for the classification of tibial plateau fractures: a systematic review

Pubmed

"Tibial Fractures"[Mesh] OR "Tibia/injuries"[Mesh] OR Tibia fracture*[tiab] OR Tibia injur*[tiab] OR Tibial fracture*[tiab] OR Tibial injur*[tiab] OR Tibial Plateau fracture*[tiab] OR (“Tibia” [Mesh] AND intra-articular fracture* [tiab]) OR (“Tibia” [Mesh] AND intra articular fracture* [tiab])

AND

("Observer Variation"[Mesh] OR Interobserver varia* [tiab] OR Inter-observer varia* [tiab] OR interobserver concordance* [tiab] OR inter-observer concordance* [tiab] OR inter-observer agreement* [tiab] OR interobserver agreement* [tiab] OR inter-observer reliabilit* [tiab] OR interobserver reliabilit* [tiab] OR interrater varia* [tiab] OR inter-rater varia* [tiab] OR interrater concordance* [tiab] OR inter-rater concordance* [tiab] OR interrater agreement* [tiab] OR inter-rater agreement* [tiab] OR interrater reliabilit* [tiab] OR inter-rater reliabilit* [tiab]) AND (classification [Mesh] OR classification* [tiab])

Embase

1. exp tibial plateau fracture/

2. tibial plateau fracture*.ab,kf,ti.

3. tibia fracture*.ab,kf,ti.

4. tibial fracture*.ab,kf,ti.

5. tibia injur*.ab,kf,ti.

6. tibial injur*.ab,kf,ti.

7. exp tibia/ AND intra-articular fracture*.ab,kf,ti.

8. exp tibia/ AND intraarticular fracture*.ab,kf,ti.

9. 1 or 2 or 3 or 4 or 5 or 6 or 7 or 8

10. exp observer variation/

11. Observer varia*.ab,kf,ti.

12. Interobserver varia*.ab,kf,ti.

13. Inter-observer varia*.ab,kf,ti.

14. interrater varia*.ab,kf,ti.

15. inter-rater varia*.ab,kf,ti.

16. observer concordance*.ab,kf,ti.

17. interobserver concordance*.ab,kf,ti.

18. inter-observer concordance*.ab,kf,ti.

19. interrater concordance*.ab,kf,ti.

20. inter-rater concordance*.ab,kf,ti.

21. observer agreement*.ab,kf,ti.

22. interobserver agreement*.ab,kf,ti.

23. inter-observer agreement*.ab,kf,ti.

24. interrater agreement*.ab,kf,ti.

25. inter-rater agreement*.ab,kf,ti.

26. observer reliabilit*.ab,kf,ti.

27. interobserver reliabilit*.ab,kf,ti.

28. inter-observer reliabilit*.ab,kf,ti.

29. interrater reliabilit*.ab,kf,ti.

30. inter-rater reliabilit*.ab,kf,ti.

31. 10 or 11 or 12 or 13 or 14 or 15 or 16 or 17 or 18 or 19 or 20 or 21 or 22 or 23 or 24 or 25 or 26 or 27 or 28 or 29 or 30

32. exp classification/

33. classification*.ab,kf,ti.

34. 33 or 32

35. 31 and 34

36. 9 and 35

Cochrane

#1 MeSH descriptor: [Tibial Fractures]

#2 MeSH descriptor: [Tibial Fracture]

#3 (Tibia fracture*):ti,ab,kw

#4 (Tibia injur*):ti,ab,kw

#5 (Tibial fracture*):ti,ab,kw OR

#6 (Tibial injur*):ti,ab,kw

#7 (Tibial Plateau fracture*):ti,ab,kw

#8 (tibial intra-articular fracture):ti,ab,kw

#9 (tibial intraarticular fracture):ti,ab,kw

#10 #1 OR #2 OR #3 OR #4 OR #5 OR #6 OR #7 OR 8 OR 9 OR 10

#11 (Observer varia*):ti,ab,kw

#12 MeSH descriptor: [Observer variation] this term only

#13 (Interobserver varia*):ti,ab,kw

#14 (Inter-observer varia*):ti,ab,kw

#15 (Interrater varia*):ti,ab,kw

#16 (Inter-rater varia*):ti,ab,kw

#17 (Observer concordance*):ti,ab,kw

#18 (interobserver concordance*):ti,ab,kw

#19 (inter-observer concordance*):ti,ab,kw

#20 (interrater concordance*):ti,ab,kw

#21 (inter-rater concordance*):ti,ab,kw

#22 (observer agreement*):ti,ab,kw

#23 (interobserver agreement*):ti,ab,kw

#24 (inter-observer agreement*):ti,ab,kw

#25 (interrater agreement*):ti,ab,kw

#26 (inter-rater agreement*):ti,ab,kw

#27 (observer reliabilit*):ti,ab,kw

#28 (interobserver reliabilit*):ti,ab,kw

#29 (inter-observer reliabilit*):ti,ab,kw

#30 (interrater reliabilit*):ti,ab,kw

#31 (inter-rater reliabilit*):ti,ab,kw

#32 #11 OR # 12 OR #13 OR #14 OR #15 OR #16 OR #17 OR #18 OR #19 OR #20 OR #21 OR #22 OR #23 OR #24 OR #25 OR #26 OR #27 OR #28 OR #29 OR #30 OR #31

#33 (classification*):ti,ab,kw

#34 MeSH descriptor: [classification] this term only

#35 #33 OR #34

#36 #32 AND #35

#37 #10 AND #36

Web of Science

#1

TS=("Tibial fracture*") OR TS=("Tibia fracture*") OR TS=("Tibia injur*") OR TS=("Tibial injur*") OR TS=("Tibial Plateau fracture*") OR TS=("tibial intra-articular fracture*") OR TS=("tibial intra articular fracture*") OR (TS=(“tibia”) AND TS=(“intra-articular fracture*)) OR (TS=(“tibia”) AND TS=(“intra articular fracture*))

#2

(TS=(“Observer varia*”) OR TS=(“Interobserver varia*”) OR TS=(“Inter-observer varia*”) OR TS=(“interrater varia*”) OR TS=(“inter-rater varia*”) OR TS=(“observer agreement*”) OR TS=(“interobserver agreement*”) OR TS=(“inter-observer agreement*”) OR TS=(“interrater agreement*”) OR TS=(“inter-rater agreement*”) OR TS=(“observer concordance*”) OR TS=(“interobserver concordance*”) OR TS=(“inter-observer concordance*”) OR TS=(“interrater concordance*”) OR TS=(“inter-rater concordance*”) OR TS=(“observer reliabilit*”) OR TS=(“interobserver reliabilit*”) OR TS=(“inter-observer reliabilit*”) OR TS=(“interrater reliabilit*”) OR TS=(“inter-rater reliabilit*”))

#3

(TS=(“classification*”)

#4

#3 AND #2

#5

#4 AND #1
